# Deep anterior lamellar keratoplasty and penetrating keratoplasty in macular corneal dystrophy: comparison of visual and topographic outcomes and complications

**DOI:** 10.5935/0004-2749.2023-0109

**Published:** 2024-03-05

**Authors:** Ayşe Tüfekçi Balıkçı, Ayşe Burcu, Züleyha Yalnız Akkaya, Evin Singar, Selma Uzman

**Affiliations:** 1 Department of Ophthalmology, Ankara Training and Research Hospital, University of Health Sciences Turkey, Ankara, Turkey

**Keywords:** Macular corneal dystrophy, Corneal dystrophies, Hereditary, Keratoplasty, Penetrating, Corneal transplantation

## Abstract

**Purposes:**

This study aims to assess and compare the postoperative visual and topographic outcomes, complications, and graft survival rates following deep anterior lamellar keratoplasty and penetrating keratoplasty in patients with macular corneal dystrophy.

**Methods:**

In this study we enrolled 59 patients (23 male; and 36 female) with macular corneal dystrophy comprising 81 eyes. Out of these, 64 eyes underwent penetrating keratoplasty, while 17 eyes underwent deep anterior lamellar keratoplasty. The two groups were analyzed and compared based on best-corrected visual acuity, corneal tomography parameters, pachymetry, complication rates, and graft survival rates.

**Results:**

After 12 months, 70.6% of the patients who underwent deep anterior lamellar keratoplasty (DALK) and 75% of those who had penetrating keratoplasty (PK) achieved a best-corrected visual acuity of 20/40 or better (p=0.712). Following surgery, DALK group showed lower front Kmean (p=0.037), and Q values (p<0.01) compared to the PK group. Postoperative interface opacity was observed in seven eyes (41.2%) in the DALK group. Other topography values and other complications (graft rejection, graft failure, cataract, glaucoma, microbial keratitis, optic atrophy) did not show significant differences between the two groups. The need for regrafting was 9.4% and 11.8% in the PK and DALK groups, respectively (p=0.769). Graft survival rates were 87.5% and 88.2% for PK and DALK; respectively (p=0.88 by Log-rank test).

**Conclusion:**

Both PK and DALK are equally effective in treating macular corneal dystrophy, showing similar visual, topographic, and survival outcomes. Although interface opacity occurs more frequently after DALK the visual results were comparable in both groups. Therefore, DALK emerges as a viable surgical choice for patients with macular corneal dystrophy without Descemet membrane involvement is absent.

## INTRODUCTION

Macular corneal dystrophy (MCD) is a type of stromal dystrophy characterized by bilateral diffuse stromal haze and scattered localized gray-white stromal opacities primarily affecting the anterior stroma in the cornea`s center and the posterior stroma in the cornea`s periphery^(1)^. Histopathologically, the condition is distinguished by the accumulation of glycosaminoglycans, which can be detected with positive staining using alcian blue, colloidal iron, metachromatic dyes, and periodic acid-Schiff, found beneath the epithelium, between stromal lamellae, within the keratocytes, and endothelial cells. The condition leads to a decrease in central corneal thickness due to changes in corneal stiffness and decreased water-binding capacity caused by the deterioration of the corneal stroma^(2)^. It is an autosomal recessive disorder associated with decreased proteoglycan synthesis often resulting from a mutation in the CHST6 gene^(3)^. MCD is more prevalent in certain regions such as India, Saudi Arabia, and Iceland^(4,5,6)^. Clinical symptoms typically manifest during the first decade of life, and affected individuals usually experience severe visual impairment by their third decade, necessitating corneal transplantation. MCD accounts for 10%-75% of corneal dystrophies that require corneal transplantation, as reported in various studies^(7)^.

Corneal stomal dystrophy often necessitates treatment trough either penetrating keratoplasty (PK) or deep anterior lamellar keratoplasty (DALK). While DALK offers certain advantages over PK, it is also associated with a higher recurrence rate, interface opacity, and potentially inferior vision compared to PK. Nevertheless, some studies have suggested that DALK, especially when performed with the large air bubble technique, and in cases of MCD without Descemet`s involvement, can yield superior safety and comparable visual outcomes to PK^(8,9,10,11)^.

The objective of this study was to conduct a comparison between the postoperative visual and topographic outcomes, postoperative complications, and graft survival rates of DALK and PK specifically in MCD patients.

## METHODS

This study was conducted at a tertiary center`s Cornea Department. The study protocol received approval from the Institutional Review Board of the hospital adhering to the principles of the Declaration of Helsinki. A retrospective review of the medical records was performed for all patients who underwent either PK or DALK for MCD at the authors’ institution between January 1990 and December 2021. Patients were included in the study if their clinical features confirmed the diagnosis of MCD, and they had a follow-up period of more than 12 months. The diagnosis of MCD was based on patient history, family history, and slit-lamp findings, which included the presence of multiple gray-white stromal opacities extending to the deep stroma and periphery, along with stromal blurring between these lesions^(1)^. Various data points were collected, including demographic characteristics of the patients, best-corrected visual acuity (BCVA), preoperative intraocular pressure (IOP), disease diagnosis age, operation age, duration of follow-up, maximum curvature power (Kmax), mean curvature power of the cornea`s front surface (front Kmean), mean curvature power of the cornea`s back surface (back Kmean), thinnest corneal thickness (TCT), apical corneal thickness (ACT), central corneal thickness(CCT), corneal volume (CV), corneal astigmatism, asphericity in the central 6 mm (Q value), complications, disease recurrence, graft survival rate, preoperative phototherapeutic keratectomy (PTK) rate, and re-keratoplasty rate. All operated eyes underwent comparison of their pre- and postoperative values. Moreover, eyes that underwent PK and DALK were compared with respect to various parameters, including BCVA, IOP, corneal topography and pachymetry values, and complication rates. Corneal topographic and pachymetric maps were obtained using a Scheimpflug imaging Pentacam (Oculus Optikgeräte, Wetzlar, Germany). For statistical analysis, the visual results were converted into logarithms of the minimum angle of resolution units. Visual acuity measurements, such counting fingers, tracking hand movements, and perceiving light were converted to 0.004, 0.002, and 0.001, respectively.

Penetrating keratoplasty procedures were performed using the conventional method. On the other hand, DALK was conducted using the big-bubble technique originally described by Anwar and Teichman. When attempts to produce a large bubble were unsuccessful, a manual dissection was carried out layer by layer. The recipient size ranged from 7.00 to 8.25 mm for eyes treated with either PK or DALK. In the PK group, it was customary to size the donor tissue trephine 0.5 mm larger than the host trephine while in the DALK group it was sized 0.25 mm larger. Closure of the donor buttons involved either a single continuous suture or 16 interrupted 10-0 nylon sutures for both groups.

Both groups underwent thorough ophthalmologic examination prior to surgery and during postoperative visits. These examinations included assessment of uncorrected visual acuity (UCVA), BCVA, manifest refraction, slit-lamp biomicroscopy, and corneal topographic analysis using the Pentacam topography system (Oculus Optikgeräte, Wetzlar, Germany). Postoperatively, the patients received treatment with topical antibiotics (moxifloxacin 0.5% QID) for 2-3 weeks, and topical steroids for a minimum of 6-9 months for those who underwent PK and at least 6 months in a gradually decreasing dosage for the eyes that underwent DALK. If there was an increase in intraocular pressure topical antiglaucoma medications were added as needed. Regular follow-up evaluations were scheduled at 1 day, 1 week, 1 month, and every 3 months for the first 2 years and annually thereafter. During the most recent follow-up visit, the UCVA, BCVA, and postoperative graft clarity were noted. Postoperative BCVA was compared based on vision levels at the 1-year mark. Corneal graft rejection can be characterized by several defining features, including the appearance of a rejection line, the spread of corneal edema, keratic precipitates restricted to a previously clear graft, and an anterior chamber reaction with reduced vision. For graft failure, the criterion used was the irreversible loss of central graft clarity regardless of the visual acuity level. To identify clinically significant recurrence, the following indicators were employed: biomicroscopic signs of recurrent disease and a decrease in BCVA to 20/40 or worse.

Statistical analyses were conducted using IBM SPSS Statistics 23.0 (IBM Corp. Released 2015, IBM SPSS Statistics for Windows, Version 23.0, Armonk, NY: IBM Corp.). The results are presented as mean ± standard deviation or median (minimum-maximum) for continuous variables. Categorical variables were described in terms of frequency and percentage. The normality of all data samples was assessed using the Kolmogorov-Smirnov test. For categorical variables, a comparison was made using the Pearson Chi-squared test. Independent samples t-test was employed for normally distributed data, while the Mann-Whitney U test was used for normally distributed independent variables that were not suitable for a t-test. To compare the pre- and post surgery values for all operated eyes, paired-samples t-test and Wilcoxon signed rank test were utilized. The comparison of complications between the two groups was performed using chi-square analysis. Graft survival curves were produced using the standard Kaplan-Meier method and log-rank test. A p-value of less than 0.05 was considered statistically significant.

## RESULTS

The study consisted of 59 patients (23 male; and 36 female), with a total of 81 eyes affected by MCD. The average diagnosis and surgery ages were 33.63 ± 8.20, and 38.79 ± 10.48 years, respectively (range, 13-51 years and 13-60 years). The mean follow-up period was 6.6 ± 3.9 years (ranging from 1 to 16 years). Out of the total eyes, 64 underwent PK, and 17 eyes underwent DALK. Following the surgery, there was an increase in Kmax, thickness parameters, and corneal astigmatism. Among eyes with MCD, there was no significant change in front Kmean and Q values after surgery, but there was a significant decrease observed in back Kmean. Table 1 provides a statistical comparison of sex, IOP, BCVA, diagnosis age, operation age, follow-up duration, and corneal topography data between the study groups. As evident from the results, there were notable differences between the age at the time of surgery, follow-up duration, front Kmean, and Q values after the operation in the eyes that underwent PK and DALK, However, there were no significant differences between the other values. Preoperative and postoperative BCVA levels did not exhibited any significant differences between the two groups. At 12 months, 12 eyes (70.6%) of the DALK group and 48 eyes (75%) of the PK group achieved a BCVA of 20/40 or better (p=0.712). Similarly, at the last follow-up, 10 eyes (58.8%) of the DALK group and 36 eyes (56.3%) of the PK group had a BCVA of 20/40 or better (p=0.849). Comparing the two groups, the DALK group had a younger age at the time of surgery (p=0.006). Additionally, after the surgery, the DALK group showed lower front Kmean (p=0.037) and Q values (p<0.01) compared to the PK group.

**Table 1:** Comparison of surgical pre-/postoperative values for certain parameters between study groups

	PK (n=64)	DALK (n=17)	p-value
BCVA preop (logMAR)	1.40 ± 0.49	1.15 ± 0.43	0.068
BCVA postop (log MAR)	0.43 ± 0.33	0.45 ± 0.39	0.823
IOP (preop)	14.34 ± 2.40	13.94 ± 2.65	0.550
Sex (female/male)	37/27	11/6	0.607^#^
Diagnosis age (year)	32.73 ± 8.26	28.73 ± 6.7	0.094
Operation age (year)	39.98 ± 10.28	32.18 ± 7.92	0.006*
Follow time (year)	7.85 ± 4.02	5.47 ± 3.35	0.018*
Pre-Kmax	49.76 ± 4.03	47.99 ± 2.57	0.330^**^
Pre-front Kmean	44.29 ± 3.82	43.30 ± 1.44	0.295
Pre-back Kmean	–5.78 ± 0.34	–5.82 ± 0.39	0.696^**^
Pre-TCT	345.95 ± 54.75	322.66 ± 55.56	0.256
Pre-ACT	389.57 ± 51.02	365.75 ± 61.16	0.239
Pre-CCT	432.33 ± 38.77	377. 42 ± 60.95	0.061
Pre-CV	46.32 ± 5.55	43.51 ± 4.81	0.183
Pre-Corneal astigmatism	3.42 ± 1.94	3.15 ± 1.53	0.695
Pre-Q value	–0.75 ± 0.48	–0.55 ± 0.36	0.251
Post-Kmax	51.92 ± 4.69	50.37 ± 3.52	0.157
Post-front Kmean	43.26 ± 3.97	40.65 ± 3.11	0.037*
Post-back Kmean	–6.35 ± 0.41	–6.32 ± 0.54	0.883
Post-TCT	513.37 ± 42.30	510.14 ± 39.42	0.811
Post-ACT	535.62 ± 46.64	530.21 ± 42.01	0.715
Post-CCT	530 ± 46.28	524.78 ± 42.41	0.729
Post-CV	56.68 ± 5.37	54.30 ± 4.85	0.171
Post-Corneal astigmatism	4.54 ± 1.95	4.45 ± 2.29	0.900
Post-Q value	–0.29 ± 0.79	–1.65 ± 1.02	<0.01*

PK= penetrating keratoplasty; DALK= deep anterior lamellar keratoplasty; BCVA= best-corrected visual acuity; IOP, intraocular pressure; Kmax, maximum curvature power; Kmean= mean curvature power; TCT= thinnest corneal thickness; ACT= apical corneal thickness; CCT= central corneal thickness; CV= corneal volume; Q value= asphericity in the central 6 mm * Independent samples t-test, ** Mann-Whitney U test, #Pearson chi-square test.

**Figure 1:**
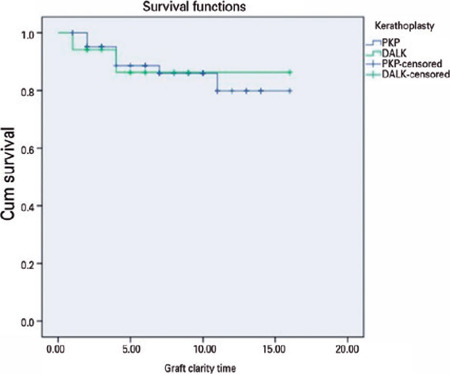
Graft survival between study groups.

The rates of PTK, complications, additional surgeries (amniotic membrane transplantation (AMT), cataract, glaucoma), re-keratoplasty rates, and graft transparency rates at the last examination were compared between the two study groups (Table 2). Complications were detected in 28 eyes (43.8%) in the PK group, and 11 eyes (64.7%) in the DALK group during the follow-up period. Notably, interface opacity, an unexpected complication after PK surgery, was observed in seven (41.2%) eyes in the DALK group. This complication contributed to a higher overall complication rate in the DALK group. It is also worth mentioning that all eyes with interface opacity following DALK were those that underwent manual dissection during the operation. However, there was no significant difference between the two groups regarding other complications (graft rejection, graft failure, cataract, glaucoma, microbial keratitis (MK), optic atrophy). MK was observed in both groups (5.9% in DALK and 9.4% in PK). In the PK group, one eye showed improvement with medical treatment, while AMT was performed in the other affected eyes. There were no instances of recurrence observed in the PK group during the follow-up period, while in the DALK group, a single eye (5.9%) experienced recurrence (p=0.051). Notably, this recurrence occurred after 16 years in the eye that underwent lamellar dissection during the DALK procedure. In the PK group, graft rejection episodes were seen in 14 eyes (21.9%), out of which 7 eyes showed improvement with intensive topical and systemic steroid therapy, while 7 eyes developed irreversible rejection. However, no graft rejections were observed in the DALK group. Re-keratoplasty was performed in six eyes (9.4%) in the PK group and in two eyes (11.8%) in the DALK group (p=0.769). Among the PK group, five eyes required re-keratoplasty due to graft rejection, while one eye required re-keratoplasty due to graft opacification after keratitis. In the DALK group, re-keratoplasty was performed in one eye due to graft failure and in one eye due to graft opacification after keratitis. At the most recent visit, graft transparency was observed in 54 eyes (77.1%) in the PK group and in 16 eyes (94.1%) in the DALK group (p=0.565). Regarding graft survival the PK group showed a graft survival rate of 87.5% (56 eyes) the most recent visit, while the DALK group had 88.2% (15 eyes) (p=0.88 by log-rank test). The difference in the rate of graft survival between the groups was not statistically significant (Figure 1).

**Table 2:** Comparison of diferent parameters between study groups

	PK (n=64)(%)	DALK (n=17)(%)	p-value*
PTK	3 (4.7)	4 (23.5)	**0.014**
Complication	28 (43.8)	11 (64.7)	0.124
Graft rejection	7 (10.9)	0	0.154
Graft failure	7 (10.9)	2 (11.8)	0.923
Cataract	24 (37.5)	6 (35.3)	0.867
Operation	15 (23.4)	2 (11.8)	0.293
Glaucoma			
Medication	6 (9.4)	1 (5.9)	0.649
Operation	1 (1.6)	1 (5.9)	0.308
MK	6 (9.4)	1 (5.9)	0.649
AMT	5 (7.8)	1 (5.9)	0.787
Interface opacity	0	7 (41.2)	**<0.001**
optic atrophy	2 (3.1)	0	0.460
Dystrophy recurrence	0	1 (5.9)	0.051
Re-keratoplasty	6 (9.4)	2 (11.8)	0.769
Graft transparency	54 (77.1)	16 (94.1)	0.565

PK= penetrating keratoplasty; DALK= deep anterior lamellar keratoplasty; PTK= photothe-rapeutic keratectomy; AMT= amniotic membrane transplantation; MK= microbial keratitis * Pearson chi-square test.

**Figure 2:**
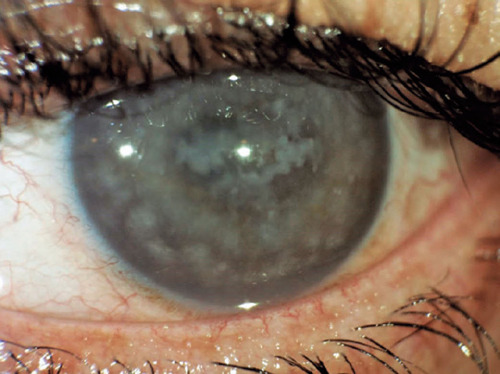
A figure of a patient with macular dystrophy.

**Figure 3:**
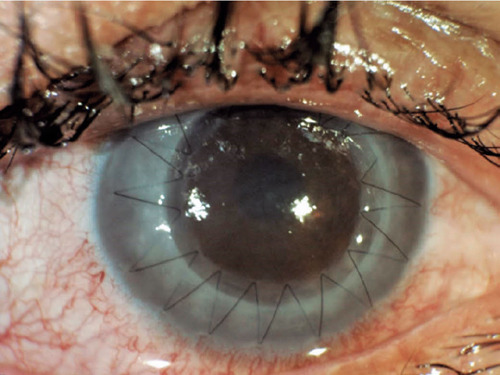
The appearance of the patient after keratoplasty.

Additionally, Figure 2 displays an image of a patient with macular dystrophy, and Figure 3 show cases a postoperative image.

## DISCUSSION

The choice of transplantation method for MCD depends on the severity of the patient’s condition. While both DALK and PK have demonstrated successful outcomes in the past, there have been concerns regarding lamellar surgery^(12,13)^. DALK surgery offers the advantage of preserving the eye’s integrity, potentially leading to lower complication rates during and after surgery. However, since macular dystrophy can also affect the deep layers of the cornea, it has been speculated that the visual prognosis following DALK might be inferior to that after PK, and some studies have supported this notion^(8)^. Nevertheless, if the deep stroma and Descemet membrane are not involved in the disease, DALK can be a suitable option. Descemet`s membrane-barricade techniques, such as the Anwar big bubble technique, have been shown in certain studies to provide visual results comparable to, or even better than, those of PK^(9,10)^.

In this study, there were no significant differences in the postoperative visual and refractive results between the DALK and PK groups, which align with the findings in the existing literature^(9,10,11)^. Postoperatively, there was a significant improvement in BCVA in all eyes. However, it is worth noting that in the study conducted by Cheng et al, the PK group exhibited better BCVA compared to the DALK group at 1, 2, 3, and 5 years^(8)^. In our series, 70.6% of eyes in the DALK group and 75% of eyes in the PK group achieved a BCVA of 20/40 or better at 12 months. At the last follow-up, 10 eyes (58.8%) in the DALK group and 36 eyes (56.3%) in the PK group achieved a BCVA of 20/40 or better. The postoperative BCVA levels of 20/40 and above after DALK and PK in the current study, along with data from previous studies reported by different institutes are presented in Table 3. It is possible that differences in surgical experience and the occurrence of intraoperative or postoperative complications could account for the varying rates of vision observed in these studies.

**Table 3:** Postoperative BCVA, graft rejection rates, and graft survival rates reported by different authors

		Postoperative BCVA (20/40 and above) (%)	Graft rejection rate (%)	Irreversible (%)	Graft survival rate (%) (last follow-up)
Our study	PK = 64	75 (1 year) 56.3 (last follow-up)	21.9	10.9	87.5
	DALK = 17	70.6 (1 year) 58.8 (last follow-up)	0	0	88.2
Cheng et al.^(8)^	PK = 57	PK>DALK (1,5 and 10 years)			87.7 87.2 (5 years)
	DALK = 21	(p<0.05)			85.7 72.7 (5 years)
Reddy et al.^(11)^	PK = 109	60 (1 year)	24.8		77
	DALK = 21	67 (1 year)	0		81
Sogutlu San et al.^(9)^	PK = 41	68.5 (last follow-up)	12.1	7.3	
	DALK = 35	70.7 (last follow-up)	8.5	0	
AlAraj et al.^(10)^	PK = 135	76.8 (last follow-up)	14	26.3	91.1
	DALK = 22	90.6 (last follow-up)	4.5	0	95.5
Al-Swailam et al.^(12)^	PK = 229	55 (last follow-up)	20.5	3.5	96 (age < 40)
	DALK = 0				75 (age > 40)
Unal et al.^(22)^	PK = 0	75.4 (All dystrophies)	4.3	0	
	DALK = 44	(last follow-up)			

PK = penetrating keratoplasty; DALK = deep anterior lamellar keratoplasty.

In this study, the PK group had a higher average age at the time of the operation. The mean follow-up period was 7.85 years for the PK group and 5.47 years for the DALK group. For many years, only PK was performed for MCD patients, but with advancements in lamellar surgery techniques, DALK also started to be performed in suitable cases. Therefore, the eyes that underwent PK had a significantly longer follow-up period.

The study also compared the topographic data of the patients. Following the surgery, there was an increase in Kmax, thickness parameters, and corneal astigmatism. However, in eyes with MCD, there was no significant change in front Kmean and Q values after surgery, but a significant decrease was observed in back Kmean. After the surgery, the DALK group exhibited lower front Kmean and Q values. However, the other values, did not show significant differences between the DALK group and the PK group. Pachymetry confirmed previous associations between MCD and central corneal thinning^(14,15)^. The cornea of MCD patients is typically thinner, leading to a significantly lower CV. While most patients with MCD do not exhibit corneal ectasia there are case studies in the literature that have linked keratoconus to MCD^(16,17,18)^. Both keratoconic and MCD corneas exhibit reduced keratan sulfate concentrations and an elevated dermatan to keratan sulfate ratio. This similarly suggests that abnormal deposits in MCD may impact the biochemistry of collagen fibril size or packing, predisposing the cornea to thinning and ectasia^(19)^.

In a previous study that evaluated corneal topography in stromal corneal dystrophies, the mean values in the MCD group were similar to the present study^(15)^. Both studies had preoperative Kmax values above 48 D. However, significant differences were noted in postoperative keratometry and pachymetry values in the current study, mainly because the donor graft used was of normal thickness and larger in size than the recipient bed. The significant difference in front Kmean values between the PK and DALK groups in the present study could be attributed to the sizing of the donor trephination, which was 0.5 mm larger than the host trephine in the PK group and 0.25 mm larger in the DALK group. The postoperative corneal suturing resulted in a significant increase in corneal astigmatism, although there was no significant difference between the two groups in this aspect. The corneal Q value reflects the change in corneal power from the center to the periphery, and changes in corneal asphericity are linked to changes in corneal curvature. In this study, the postoperative Q value was notable lower in the DALK group. Previous research has demonstrated that higher visual function was associated with a more prolate corneal shape and smaller postoperative Q values^(20)^.

PTK can restore vision and potentially delay or eliminate the need for invasive surgical procedures in suitable cases. This is particularly crucial for corneal dystrophies like MCD that typically manifest in childhood and adolescence. PTK has been shown to be an efficient method for vision restoration in patients with MCD, particularly in young populations, allowing for the postponement of corneal transplantation. However, in some cases, particularly in patients who are presbyopic or hyperopic prior to the treatment, PTK for MCD may lead to hyperopic shift, which could result in unsatisfactory outcomes despite a clearer visual axis ^(21)^. In this study, three eyes (4.7%) in the PK group and four eyes (23.5%) in the DALK group had a history of preoperative PTK. It can be inferred that the rates of PTK were lower in this group, given that the patients who underwent PK were diagnosed at a later stage and had more advanced dystrophy.

Table 3 presents the postoperative graft rejection and graft survival rates after DALK and PK in the current study, as well as those reported by different authors. In this study, irreversible graft rejection was observed in seven (10.9%) eyes after PK, while no such cases were observed in the DALK group. Although the graft rejection rate was higher after PK, this difference was not statistically significant. Graft failure occurred in seven (10.9%) eyes after PK and in two (11.8%) eyes after DALK. Additionally, re-keratoplasty was performed in six (9.4%) eyes after PK and in two (11.8%) eyes after DALK. There was no significant difference between the two groups. Overall, the rates of graft rejection and re-keratoplasty were found to be higher in studies focused on PK ^(8,9,10,11,12,22)^. During the most recent visit, the graft survival rates were 87.5% (56 eyes) in the PK group and 88.2% (15 eyes) in the DALK group. Central graft transparency was observed in 54 eyes (77.1%) in the PK group and in 16 eyes (94.1%) in the DALK group at the last visit. Despite differing results in other studies, no significant differences were found between the PK and DALK groups regarding graft survival^(8,10,11,12)^.

In this study, the rates of cataract, glaucoma, and MK, along with corresponding surgical treatments for these complications, were similar in both groups. In the PK group, the most common complications were graft rejection, cataract, and glaucoma, while in the DALK group, they were cataract and interface opacity. Eyes that underwent manual dissection during the DALK procedure experienced interface opacity. Similarly, in the study by AlAraj et al.^(10)^, the most common complications in the PK group were rejection, cataract, and MK, while in the study by Reddy et al.^(11)^, they were secondary glaucoma, MK, and endophthalmitis. Both DALK and PK groups in the current study showed cases of MK (5.9% in DALK and 9.4% in PK). In the PK group, one eye improved with medical treatment, while in the other eyes, AMT was performed. The literature indicates that the incidence of MK after PK can be as high as 11.9%^(23)^. No dystrophy recurrence was observed in the PK group, whereas in the DALK group, recurrence was observed in one eye after 16 years. In contrast, the study by AlAjar et al. reported clinically significant recurrence in one eye (4.5%) in the DALK group after 5.1 years and in four eyes (2.9%) in the PK group over a mean interval of 9.6 ± 5.1 years^(10)^.

The main limitation of this study is that the number of patients who underwent PK was higher than those who underwent DALK. This was because the dystrophy generally progressed to the deep corneal layers by the time the patients sought treatment, and PK was more frequently performed in previous years. Despite this imbalance in patient numbers, the comparison between the two groups was done concurrently to ensure unbiased outcomes. Additionally, endothelial counts were not evaluated in this study due to insufficient data availability.

In conclusion, both PK and DALK demonstrate comparable visual, topographic, and survival outcomes in treating MCD. This study found similar postoperative complications and graft survival rates in eyes undergoing PK and DALK. Although the PK group had a slightly higher graft rejection rate, both groups achieved comparable graft survival rates with appropriate treatment. Interface opacity emerged as a more common complication after DALK in cases where manual dissection was performed during the operation, but the visual outcome remained similar in both groups. Therefore, DALK surgery presents a suitable surgical option for patients with MCD without Descemet membrane involvement.
